# A description of preimaginal stages of *Pseudaspidapion botanicum* Alonso-Zarazaga & Wang, 2011 (Apionidae, Curculionoidea)

**DOI:** 10.3897/zookeys.260.4450

**Published:** 2013-01-21

**Authors:** Zhiliang Wang, M. A. Alonso-Zarazaga, Dakang Zhou, Runzhi Zhang

**Affiliations:** 1 Key Laboratory of Zoological Systematics and Evolution, Institute of Zoology, Chinese Academy of Sciences (IZCAS), Beijing, China; 2 Depto. de Biodiversidad y Biología Evolutiva, Museo Nacional de Ciencias Naturales (CSIC), José Gutiérrez Abascal, 2, E-28006Madrid, Spain; 3 Beijing Botanical Garden, (BBG), Beijing, China

**Keywords:** Apionidae, *Pseudaspidapion botanicum*, larva, pupa, morphology, biology

## Abstract

The preimaginal stages including egg, mature larva and pupa of *Pseudaspidapion botanicum* Alonso-Zarazaga & Wang, 2011 were described and figured, diagnostic characters of larva and pupa were discussed, and corresponding biological information was supplied. The nomenclature of frontal setae in the larva compared with curculionid weevils, the absence of the hypopharyngeal bracon in the larva, and the metafemoral setae in the pupa were discussed. Common and different characters among the larvae of *Pseudaspidapion botanicum*, *Aspidapion radiolus* (Marsham, 1802) and *Aspidapion aeneum* (Fabricius, 1775) were also provided.

## Introduction

*Pseudaspidapion botanicum* Alonso-Zarazaga & Wang, 2011 belongs to the tribe Aspidapiini Alonso-Zarazaga, 1990. There are 6 species of the genus *Pseudaspidapion* Wanat, 1990 recorded from China, 2 of which are only known from female specimens, and almost all of which were described without biological information apart from *Pseudaspidapion botanicum* (Alonso-Zarazaga et al., 2011). Additionally, no species of *Pseudaspidapion* have been previously studied for developmental stages.

The knowledge of weevil larvae is still very low compared with that of the adults because of a variety of reasons (Marvaldi 1999). Authors have sparsely referred to apionid larvae in detail (e.g., Gosik et al. 2010; Sanz Benito and Gurrea Sanz 1999; Bennett 1992; May 1994), and most of the corresponding literature has only simple descriptions or keys for identification of species or higher taxa of Curculionoidea (Emden 1938; Marvaldi 1997, 2005; May 1993). Furthermore, it is rare for authors to have elaborated on the developmental stages of species of Aspidapiini. Thus far, only *Aspidapion radiolus* (Marsham, 1802) and *Aspidapion aeneum* (Fabricius, 1775) have been mentioned in a species key by Emden (1938).

Therefore, in order to supplement to the available data of *Pseudaspiapion* for distinguishing this oriental genus from other lingeages in Aspidapiini (*Aspidapion* Schilsky, 1901, *Flavopodapion* Korotyaev, 1987 and *Harpapion* Voss, 1966) (Alonso-Zarazaga 2011), we collected larvae and pupae from the host plant and document here its preimaginal stages. Moreover, relevant biological records of *Pseudaspidapion botanicum* were also provided.

## Materials and methods

Specimens examined of larvae and pupae are deposited in the Institute of Zoology, Chinese Academy of Sciences, Beijing (IZCAS).

Descriptions were made and photographs were taken with a CCD Qimagine MicroPublisher 5.0 RTV mounted on a Zeiss SteREO Discovery. V12 microscope. Extended focus images were generated with Auto-Montage Pro 5.03.0061 and edited with Adobe Photoshop CS5 if required. Microscopic slides were studied under a Leica DM 2500 microscope and photos were taken with a Nikon CoolPix 5400. Drawings were made from the original photographs by using the software Adobe Illustrator CS5, or directly by using a drawing tube attached to the microscope.

Nomenclature of the larval chaetotaxy mainly follows Marvaldi (1999) and May (1993, 1994) (some differences were pointed out in discussion below), and that of the pupa mainly follows Gosik et al. (2010). The dissecting method used follows May (1979, 1993). Indistinct structures were pigmented by “Chlorazol Black E” for further examination. After description, all structures of each individual were put into glycerin vials (1.8ml) to remain together with the adult specimens.

## Description

**Egg**: round, yellowish to white, diameter ca. 0.2–0.3mm.

**Mature larva**: Meaurements (mm): Body length: ca. 2.0–3.0, width: ca.1.0–1.5; Capsule length (in front view): ca. 0.4–0.45, width ca. 0.4–0.44.

**Figures 1–2.**
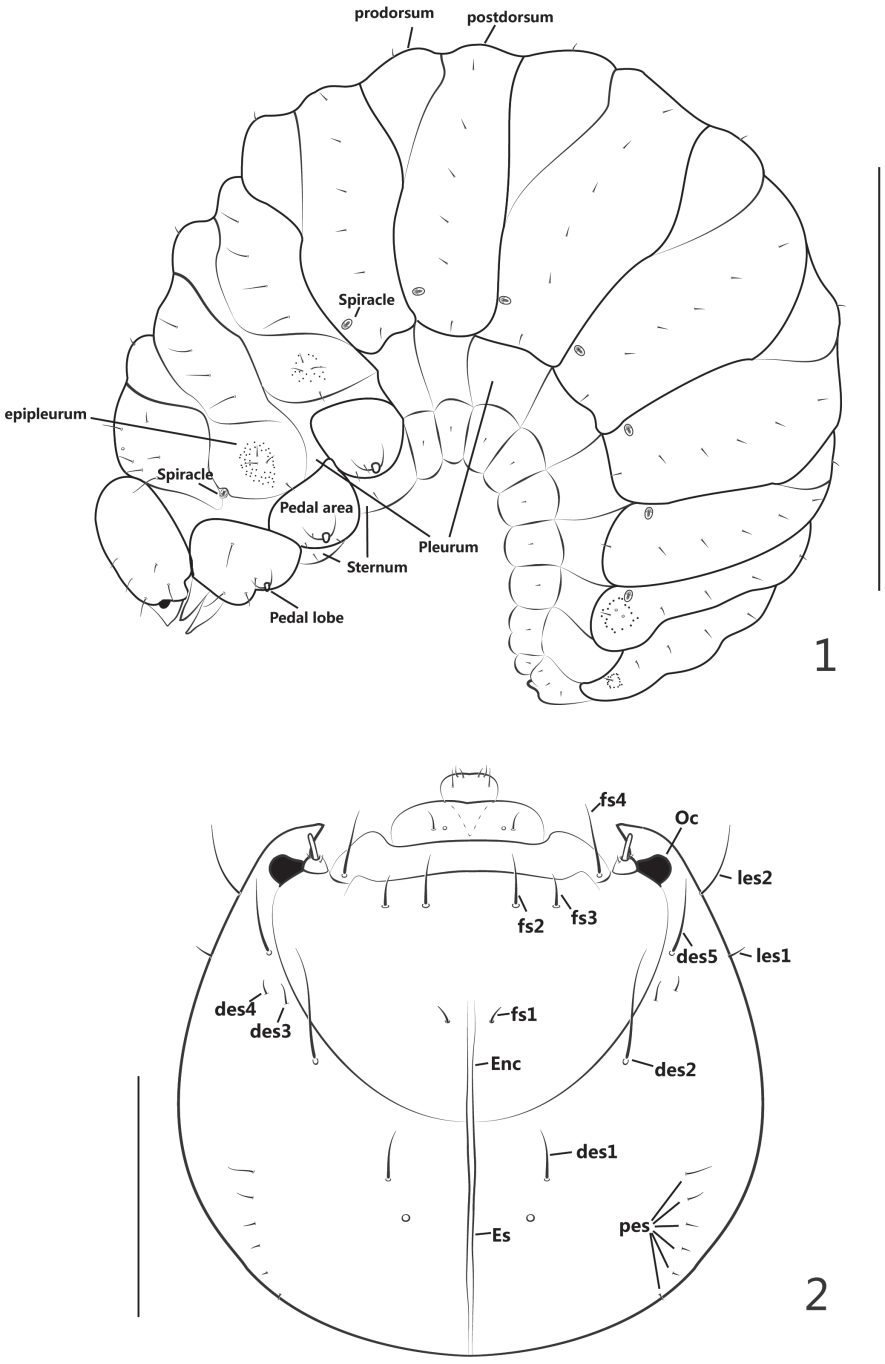
Larva. **1** mature larva, lateral view **2** head, dorsal view. Scales: **1**: 1mm. **2**: 0.2mm.

**General appearance** (Figure 1): Body plump and distinctly curved (C-shaped), with a large quantity of white fat inside, sub-cylindrical, with a comparatively small, yellowish to pale brown head; cuticle minutely spiculate, without visible pigmented, sclerotized areas; body segments with very short setae, pedal lobes in conspicuous knobs.

**Figures 3–9.**
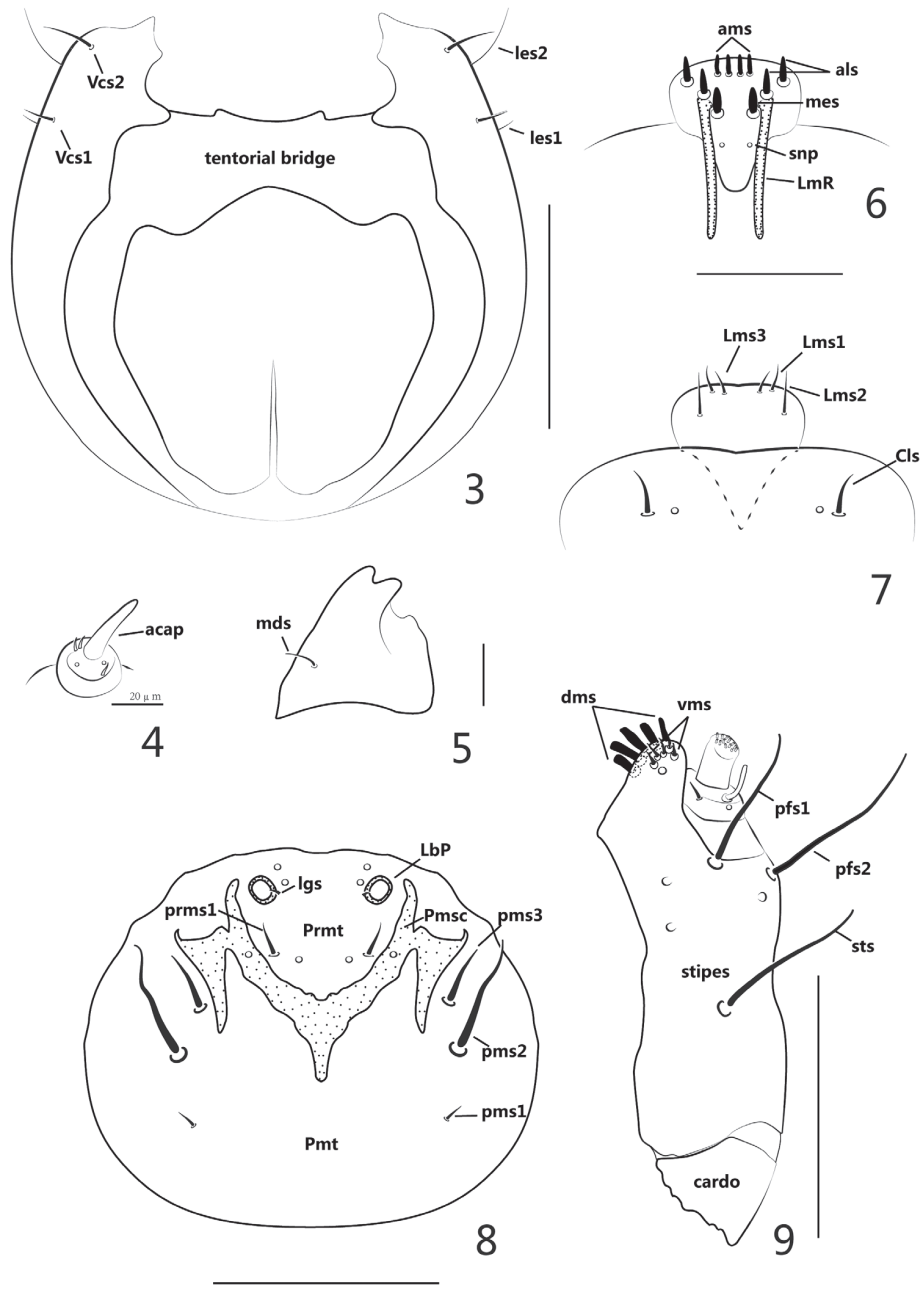
Larva. **3** head, ventral view **4** antenna **5** left mandible **6** epipharynx **7** labrum and clypeus **8** labium **9** right maxilla. Scales: **3**: 1mm. **4**: 0.02mm. **5**: 0.05mm. **6–7**: 0.05mm. **8–9**: 0.1mm.

**Head** (Figures 2–3) moderately retracted within prothorax, epicranial line distinct, undivided, wide; frontal lines distinct, narrow, completely extended to mandibular joint; endocarina short, extended to 1/2 the length of frons; epicranium with 2 pairs of lateral setae (*les*), *les1* short, *les2* long, more than 3 times longer of *les1*; dorsal epicranium with 5 pairs of setae (*des*), *des1* short, about half as long as *des2*, *des3* and *des4* much shorter, des4 a bit longer than des3, and *des5* as long as *des2*; posterior epicranium with 6 pairs of setae (*pes*), *pes1* shortest, *pes2-6* gradually longer than the former one; frons with 4 pairs of setae (*fs*), *fs1* very short, adjacent to end of epicranial line, *fs2-3* located near epistoma, nearly transversely aligned, *fs2* longer than *fs3*, *fs4* laterally positioned at epistoma close to antennae, about 1.5 × as long as *fs2*; ventral epicranium with 2 pairs of lateral setae (*Vcs*), correspondingly situated near *les*; postoccipital condyles absent, tentorial bridge wide with 2 small but moderately acute anterior projections and 2 large, obtuse-angled posterior projections; hypopharyngeal bracon absent; clypeus transverse, bearing 1 pair of setae (*cls*), inner side of *cls* bearing 1 pair of sensilla; antenna reduced to 1 article (Figure 4), with rod-like accessory sensory appendage (*acap*) more than 3x as long as wide, with 3 spinose projections and 2 sensilla; ocellus present, evidently projected, externally close to antenna.

**Mouthparts** (Figures 5–9): labrum (Figure 7) sub-semicircular, with 3 pairs of setae (*Lms*), *Lms1* and *Lms3* short, close to distal margin of labrum, *Lms2* distinctly longer and centrally localized; epipharynx (Figure 6) with 2 long, stout lateral rods (*LmR*) surpassing the suture between epistoma and clypeus, 2 pairs of anterolateral setae (*als*), 2 pairs of anteromedian setae (*ams*), 1 pair of median setae (*mes*), all epipharyngeal setae mentioned above stout, short and apically rounded, 1 pair of epipharyngeal sensory pores (*snp*) between pair of *LmR*; mandibles asymmetric, left mandible (when mouthparts viewed anteriorly) (Figure 5) apically bidentate, about as long as wide, cutting edge with 1 weakly prominent, rounded tooth, its laterodorsal surface with 1 mandibular seta (*mds*), without visible sensilla, right mandible with cutting edge nearly straight; labium (Figure 8) almost membranous except sclerotized area; labial palpus (*LbP*) vestigial, its apex a bit higher than surface of labium, like two big socket pores; premental sclerite (*Pmsc*) distinctly dilated, “Y” shaped, with 1 pair of sensilla, outer margins of *Pmsc* with exteneded sclerotized piece, irregularly triangular; ligulate area with 1 pair of tiny setae (*lgs*) and 2 pairs of sensilla; prementum (*Prmt*) with 1 pair of setae (*prms*) and 1 pair of sensilla, *prms*1 long and stout, *prms*2 quite short, close to inner side of labial palpi; postmentum (*Pmt*) with 3 pairs of setae (*pms*), *pms1* shortest and fine, *pms2* longest and stout, *pms3* a bit shorter than pms2; maxillary palpus (*MxP*) (Figure 9) with 2 segments, basal segment with 1 long and basally curved accessory process, 1 short inner seta, 1 sensillum close to accessory process, apical segment cylindrical and apically flattened with dense crenulate setae; mala with 5 dorsal robust setae (*dms*) and 4 shorter, more acute ventral setae (*vms*), 1 sensillum; stipes (*st*) bearing 1 stipital setae (*sts*), 2 palpiferal setae (*pfs*) and 3 sensilla, all 3 setae extremely thick and long, *sts* basally medioventral, *pfs1* apically medioventral, *pfs2* lateroventral; cardo completely divided from stipes.

Setae of thorax and abdomen (Figure 1) described for one side only.

**Thorax**: pronotal shield simple without fold, unsclerotized; meso- and metanotum each with 2 folds, divided into prodorsum (*fold1*) and postdorsum (*fold2*); spiracle laterally intersegmental between pro- and mesothorax, bicameral; prothoracic epipleurum indistinct, meso- and metathoracic epipleura with outline distinct, centrally tuberculate around setae; pedal area well-defined, pedal lobe (papillae) present, 2-segmented; pronotum with 6 setae (*pns*): 4 longitudinally aligned and close to anterior margin, another 2 nearly at middle area; meso- and metanotum both with 9 setae: prodorsum with 1 short setae (*prs*), epipleurum with 4 setae, 3 setae surrounded by numerous small, tuberculate projections, another situated close to pleurum; postdorsum with 4 relatively longer setae (*pds*), longitudinally aligned; pedal area with 3-4 setae; sternum with 1 tiny seta.

**Abdomen**: tergites I-VI with 2 folds, prodorsum(*fold1*) with 1 seta on disc, postdorsum(*fold2*) with 6 setae, shorter than thoracic setae and longitudinally aligned; tergites VII-VIII undivided with 5 setae, longitudinally aligned, basal 1 seta surrounded with a circle of sparse tubercles; tergite IX undivided and greatly reduced with 3 setae; 7 spiracles present, size similar, bicameral, anterolaterally located on tergites I-VII, respectively; pleura I-VIII without setae, each sternum with 1 seta.

**Figures 10–12.**
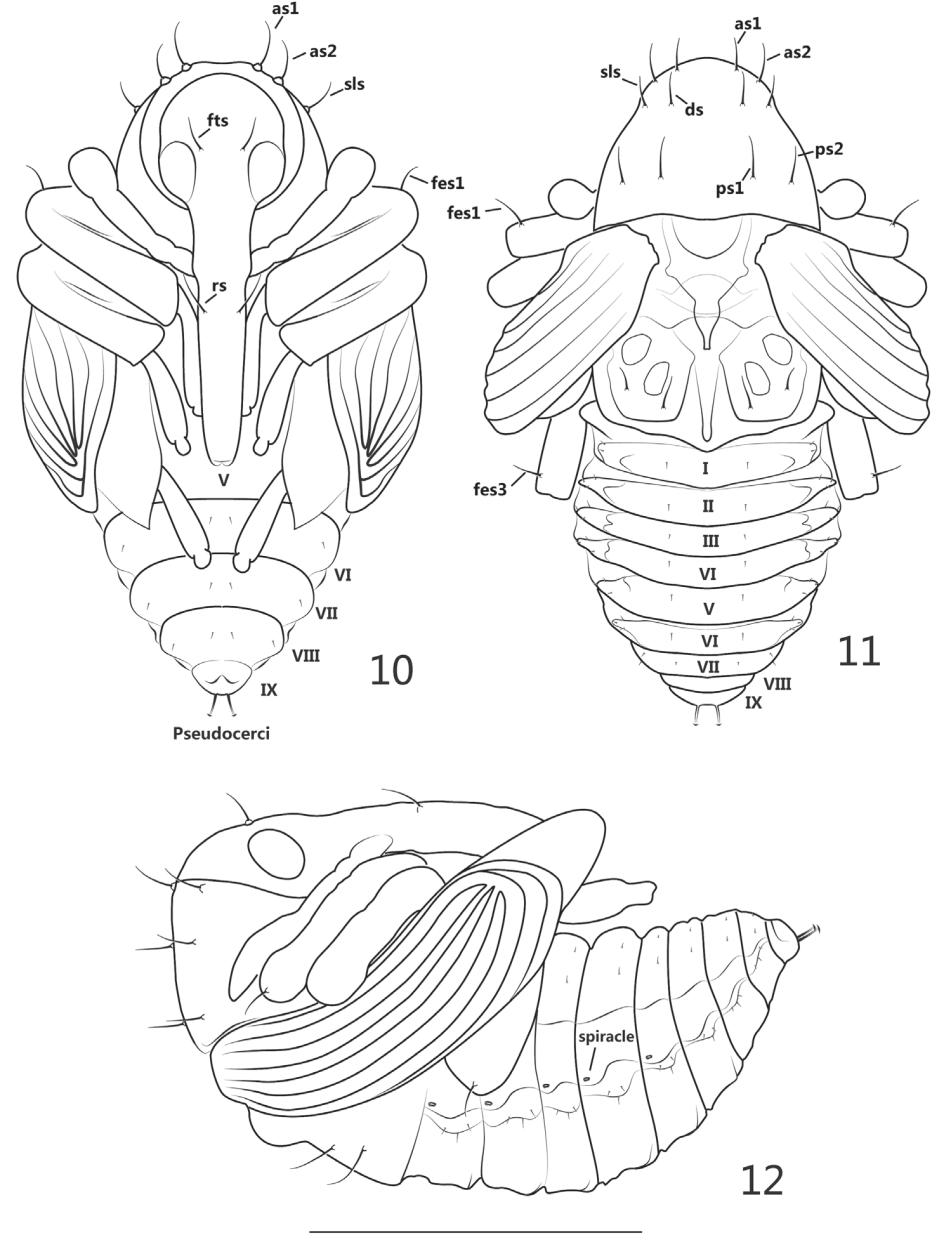
pupa. **10** ventral view **11** dorsal view **12** lateral view. Scales: **10–12**: 1mm.

**Pupa** (Figures 10–12): Measurements (mm): length: ca. 1.9–2.0, width: ca. 1.3.

**General appearance**: theca transparent with semitransparent setae, setae greatly reduced in number, inner body pure white.

**Rostrum**: in ventral view, apex reaching ventrite V, mesorostrum visibly dilated, mandibular theca weakly projected; 1 pair of rostral setae (*rs*) positioned in front of antennal insertion. Head: frons with 1 pair of setae (*fts*), about as long as rostral setae, situated at the level of hind margin of eyes; antennae basally situated near prosternum and apically extended to propleurum, sub-parallel to protibia. Thorax: in dorsal view, pronotum with 2 pairs of apical setae (*as*), 1 pair of sublateral setae (*sls*), 1 pair of discal setae (*ds*) and 2 pairs of posterolateral setae (*pls*), these long, stout and similarly elongated setae forming 2 longitudinal rows on pronotum, 3 positioned in each row and transversely localized; in ventral view, *as1*, *as2* and *sls* completely visible, distinctly longer than *vs* and *fas*; mesonotum without setae; metanotum bearing 2 pairs of setae near lateral and hind margins of metanotum, respectively, subequal in length to pronotal setae. Legs: in ventral view, metatibiae and femora covered by pterothecae, front and middle legs and meta-tarsomere visible, in lateral view, pro- and mesofemora both covered by pterothecae; pro- and metafemora apically bearing 1 slightly outcurved seta; each seta mentioned above arising from a tuberculate base. Abdomen: in ventral view, ventrites V-IX visible, in dorsal view ventrites I-VI about equal in length and width, ventrites VII-IX clearly and gradually reduced; 5 spiracles present, positioned on pleura I-V, bicameral; peudocerci narrow, elongate and slightly outcurved in ventral and dorsal views, subterminally positioned at abdominal segment IX, a bit shorter than femoral setae, apex lenticular; in ventral view, the completely visible ventrites VI-VIII with 2 pairs of setae on each segment, 1 pair situated medially, another pair situated laterally; in dorsal view, tergites I and VII each bearing 3 pairs of setae, 1 pair situated medially, 2 pairs situated laterodorsally; tegites II-VI each bearing 4 pairs of setae, 1 pair situated medially, 3 situated laterodorsally; tergites VIII-IX without clearly visible setae; all abdominal setae short and thin.

**Figures 13–16.**
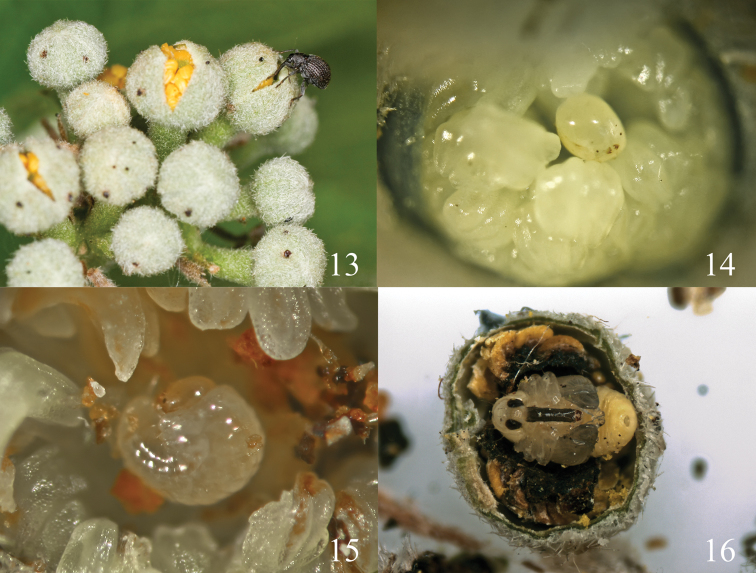
**13** the buds of *Grewia biloba* **14** egg positioned among stamens in the bud **15** first instar larva **16** pupa.

**Biological information**: *Pseudaspidapion botanicum* was collected from *Grewia biloba* G. Don var. *parviflora* (Bunge) Hand.-Mazz (Malvaceae: Grewioideae). The adults feed on leaves and buds of their host while they mate and oviposit in the bud (Alonso-Zarazaga and Wang 2011). After dissection of some 300 buds, we have found that 1-4 oviposition holes can be found on one bud surface, but only 1–2 eggs or larvae per bud are normally found, and 3 or more eggs or larvae were quite rare (Figures 13–15). The mature larva almost completely consumes the internal organs of the bud and then constructs a chamber for pupation (Figure 16), so those buds parasitized by *Pseudaspidapion botanicum* will never blossom. In normal conditions, *Pseudaspidapion botanicum* can seriously reduce the fructification rate of *Grewia biloba*, but the damage done seems not to seriously harm the plant’s reproductive rate, and the vegetative growth is not decreased by feeding on other areas. Furthermore, in late July, most of the adults disappear from their host and migrate to some other plants in sunny areas. The same case has been reported by other authors, for example, Ehret (1983) reported that apionid weevils sought shrubs and tree crowns to avoid unfavorable weather when growth of their host plants ceased temporarily or permanently, whether during the growth season or at the end of it. We have not yet had an opportunity to study the overwintering behavior of this species.

## Discussion

The diagnostic characters of the preimaginal stages of *Pseudaspidapion botanicum* can be summarized as follows. Larva: (1) antennae with *acap* more than 3× as long as wide, 3 spinose projections and 2 sensilla (2) head with 10 *des*, 8 *fs*, 4 *les*, 12 *pes* and 4 *vcs*; (3) labrum with 6 *lms*; (4) epipharynx with 4 *ams*, 4 *als*, 2 *mes* and 2 *snp*; (5) maxilla with 1 *stps*, 2 *pfs* and 3 sensilla; (6) mala with 5 *dms* and 4 *vms*; (7) each side of *Pmsc* extended outward with 1 irregularly triangular sclerotization; (8) labial palpus (*lbp*) vestigial, pore-shaped; (9) hypopharyngeal bracon absent; (10) thoracic and abdominal spiracles 1+7 in number, both bicameral. Pupa: (1) the number of setae significantly reduced on rostrum (2*rs*) and head (2*fts*); (2) pronotum with 4*as*, 2*sls*, 2*ds*, 4*ps*; (3) only metanotum with 4 setae; (4) pro- and metafemoral apical setae present, mesofemoral setae absent; (5) in lateral view, ventrites I-V each with 1 spiracle.

In terms of Anderson (1947) and Marvaldi (1999), the frontal area of Curculionidae should bear 5 setae on each side, and *fs*4 is present consistently with a consistent position and is as long as or longer than any other frontal setae. However, compared with Anderson’s and Marvaldi’s drawings, if *fs*2 of *Pseudaspidapion botanicum* is here actually “*fs*4”, then there will be an “*fs*6” (*fs*4 in this paper) and “*fs*4” will be much shorter than “*fs*6”. Hence, this situation is confused with Marvaldi’s and Anderson’s nomenclature, because their research objectives did not include apionid species. Thus, we decided to sequentially denominate the frontal setae from vertex to mandible for easy comparison of the categories within Apionidae. Moreover, frontal setae of most species are reduced from modal numbers in Curculionidae on the basis of May (1993), Emden (1938), Bennett (1992), Sanz Benito and Gurrea Sanz (1999) and Gosik et al. (2010); however, *Pseudaspidapion botanicum* is nearly undifferentiated (*fs*1-4) compared with derived curculionid species. Also, Emden (1938) thought that “frontal setae of *Apion* can be judged satisfactorily only in specimens in exactly the same position”, but at least the frontal setae of *Pseudaspidapion botanicum* are present consistently in species.

Furthermore, most authors did not mention whether mandibles are symmetric or not, and few indicated the direction of the mouthpart, which is particularly important when the mandibles are asymmetric. Inconsistent terms of morphological position resulted in those instances when “left” was identified by looking toward the body center (= proximal view) rather than away (= distal or cranial view). To avoid such ambiguities, we suggest that the left mandible should be specified and described as seen from the body center to the anterior in dorsal view.

The hypopharyngeal bracon is a bar connecting the left and right sides of the hypopharyngeal margins and supporting the hypopharynx. The presence of the bracon is one of the characters separating curculionoid larvae from those of Bruchidae and Chrysomelidae (Lawrence 1991); however, we have been unable to find this structure in *Pseudaspidapion botanicum*, and surprisingly, no author hitherto has described any species of Apionidae or even Curculionoidea as lacking it. Most authors did not refer to ventral characters of the epicranium at all when describing apionid larvae (Emden 1938; Sanz Benito and Gurrea Sanz 1999; Bennett 1992; Gosik et al. 2010), but the hypopharyngeal bracon is at least distinctly present in *Neocyba* sp. (May 1993). Obviously, more materials urgently need checking for this problem to be resolved.

It is also worthy to stress that the absence of mesofemoral setae in the pupae of Apionidae has not been recorded before, while some or all femoral setae of some groups of Anthribidae, Brentinae and Curculionidae could be absent (May 1994). It should be a useful character to distinguish *Pseudaspidapion botanicum* from other recorded pupae (*Exapion* spp., *Apion soleatum* (Wagner), *Diplapion confluens* (Kirby, 1808)).

Additionally, Emden (1938) keyed out 24 species of apionid larvae, including 2 species of *Aspidapion* (*Aspidapion radiolus* and *Aspidapion aeneum*), but all of the species were then under the genus *Apion* and not generically well-arranged in the key. The common features and several differences among *Pseudaspidapion botanicum*, *Aspidapion radiolus* and *Aspidapion aeneum* can still be extracted as in Table 1 below; however, the generic characters to verify the phylogenetic relationship between *Aspidapion* and *Pseudaspidapion* require more studies of preimaginal stages of these two genera in the future.

**Table 1.** Character comparison among *Aspidapion aeneum*, *Aspidapion radiolus* and *Pseudaspidapion botanicum*.

**Species**	***Aspidapion aeneum***^1)^	***Aspidapion radiolus***^1)^	***Pseudaspidapion botanicum***
head	as long as wide	wider than long	as long as wide
antennal accessory sensory appendage (*acap*)	slender, more than 2 × as long as wide	slender, more than 2 × as long as wide	rod-like, more than 3 × as long as wide
endocarina	long	long	short
left mandibular cutting edge	with a rather distinct tooth	with a strong, rounded dilatation	with a weakly prominent, rounded tooth
setae of labrum (*lms*)	4 pairs	4 pairs	3 pairs
anterolateral setae of epipharynx (*als*)	3 pairs	3 pairs	2 pairs
*prms* (=ventral bristles of prementum)	as close together as inner basal end of palpi	as far apart as inner basal end of palpi	as far apart as inner basal end of palpi

^1)^ Emeden’s key (1938)
